# Animal-Inclusive Protection Order Statutes: A Powerful Tool for Addressing and Mitigating Coercive Control

**DOI:** 10.1177/10778012251338405

**Published:** 2025-05-06

**Authors:** Amy J. Fitzgerald

**Affiliations:** 1 Department of Sociology and Criminology and the Great Lakes Institute for Environmental Research, University of Windsor, Windsor, ON, Canada; 2 Brooks McCormick Jr. Animal Law & Policy Program, Harvard Law School, Cambridge, MA, USA

**Keywords:** gender-based violence, intimate partner violence, animal abuse, protection order, coercive control

## Abstract

Given mounting evidence that animal abuse and intimate partner commonly co-occur, and that victims/survivors will delay leaving abusers due to concern for their companion animals, many U.S. states have amended their protection order statutes to enable the inclusion of animals. These amendments have received limited attention, and research has not examined their use and interpretation in the courts. Addressing this gap, this article analyzes legal judgments where these statutes have been cited to date, 38 in total. The findings indicate these statutes are providing much-needed protection for victims/survivors, particularly from ongoing coercive control. Recommendations for extending these protections are provided.

## Introduction

In 2014, a protection order was issued to Robin Fenty in New York to protect her from a stalker. The stalker, who had sent hundreds of pages of letters to her personal address, had also been observed outside of her home. In the letters, he claimed he and Fenty were “meant to be together.” Even more troubling, he made references to gang rape, a house on fire with a girl inside, and asked on more than one occasion, “am I scaring you tonight?” (Kevin M. v. S.B. Psychiatric Center, 2014, p. 3). He also threatened to violently disrupt the Black Entertainment Television awards, which Fenty was known to attend. One of the richest musicians of all time (Forbes, 2023), Fenty performs under her middle name: Rihanna.

The case is relatively unique, not because a celebrity was involved, but because Rihanna was able to—and did—have her companion animals included in the protection order. Citing the protection order statute language, the court ordered the accused “permanently refrain from intentionally injuring or killing without justification any pets or companion animals owned or possessed by ROBYN FENTY and any members of said protected person's family or household” (p. 9). Had the case occurred just a few years earlier,^1^ she would have been unable to include her companion animals in the protection order.

New York is among 40 other states and the District of Columbia that, in recent years, have amended their protection order statutes to enable the inclusion of (companion) animals. They have done so in response to a growing body of research indicating that intimate partner violence (IPV) and animal abuse frequently co-occur, that the presence of animal maltreatment is associated with more severe physical, psychological, and sexual violence, and that IPV victims/survivors report delaying ending abusive relationships specifically out of concern for what will happen to their companion animals (for a detailed review of the literature, see Fitzgerald, Barrett, Stevenson, & Timmons Fritz, 2021). The reasons for these concerns are many. Notably, many domestic violence (DV) shelters do not have programs in place for companion animals (on-site or off-site), although there has been a dramatic increase in program offerings in recent years (see Barrett et al., 2025). Moreover, animals are legally considered property. Therefore, the party who can demonstrate ownership would be entitled to keep the animal. This can be a source of concern when a human abuse victim/survivor cannot demonstrate ownership of an animal who they are nonetheless attached to and concerned about.

Animal-inclusive protection order statutes were introduced in an attempt to mitigate these issues and provide enhanced safety for human and animal IPV victims. To date, however, only one study has examined the extent to which animal-inclusive protection order statutes are being used. The researchers found that only the state of New York provides disaggregated statistics for issued protection orders that enables identifying the number that have included companion animals. Analysis of the data indicates that the number of protection orders issued that included companion animals grew modestly in the first 3 years (2007–2009) after the protection order statute was amended to enable the inclusion of companion animals and increased fairly steadily between 2010 and 2020 (the last year analyzed; Randour et al., 2024). Due to the limitations associated with accessing data from states on the issuance of animal-inclusive protection orders, the usefulness of these orders could not be assessed that way. Yet, given the scale of violence and harms perpetrated against both woman-identified individuals and animals, understanding if the inclusion of animals in protection orders is helpful, and if the utility varies by the types of protections enabled by the statute wording, is important. The current paper therefore addresses this gap in the literature via the following research questions: (a) Is there evidence in judgments that the courts are taking the inclusion of animals in protection orders seriously and viewing animal abuse as interconnected in important ways with the abuse of people? (b) Are animal-inclusive protection order statutes being interpreted in a manner that provides protection for human and animal victims of IPV, and gender-based violence more generally, particularly from coercive control? To address these questions, written judgments citing protection order statutes vis-à-vis animals (38 cases in total) are analyzed.

The next section provides a detailed description of the problem these protection order statutes were designed to address, with specific focus on empirical studies, followed by scholarship detailing the development of animal-inclusive protection order statutes. Next, the specific and pernicious problem of coercive control, and how it intersects with animal maltreatment, is discussed. The methods used in this study are then detailed, followed by a description of the findings. The paper closes with a discussion of key findings and recommendations aimed at better assisting human and animal victims/survivors of abuse.

## The Co-Occurrence of Animal Abuse and Gender-Based Violence

The IPV literature has for decades contained references to the presence of animal maltreatment in the context of IPV. Historically, however, animal abuse was addressed as anecdotal and contextualized as a form of property destruction (Ganley, 1985; Pence & Paymar, 1993; Walker, 1979). In the 1990s, feminist philosopher Carol Adams (1994, 1995) began highlighting animal abuse in conjunction with IPV and child abuse as critically important and worthy of focused attention, not merely as a tangential form of property abuse. Research attention followed.

Most early studies focused on DV shelters in single locations in the United States. Those early studies reported between 25% and 86% of women sampled reported their abuser also harmed/threatened their companion animals (e.g., Ascione, 1998; Carlisle-Frank et al., 2004; Faver & Cavazos, 2007). A subsequent study using the validated Partners’ Treatment of Animals Scale (Fitzgerald et al., 2016) with a sample of women in 17 DV shelters across Canada, documented an 89% co-occurrence between animal maltreatment and IPV (Barrett et al., 2020).

Despite the generally high co-occurrence between IPV and animal abuse reported in these studies, it was unclear if it was greater than what would be reported by individuals not in abusive relationships. To make that comparison and assess if animal abuse is truly linked to IPV instead of being more endemic, two studies included comparison samples. They found that IPV survivors were statistically much more likely to report perpetration of animal abuse by their partners than those in IPV-free samples (Ascione et al., 2007; Volant et al., 2008). Moreover, a study of a sample of men arrested for IPV found that 41% of the men had engaged in animal maltreatment, compared to only 1.5% of men in the general population (Febres et al., 2014).

Nonetheless, it remained unclear if the co-occurrence between animal abuse and IPV could be generalized to abusive relationships where the victim was not in a DV shelter. This is an important consideration given that most do not go to DV shelters (Barrett & St. Pierre, 2011). To address this question, Fitzgerald et al. analyzed nationally representative data from the Canadian General Social Survey. They found statistically significant relationships between animal abuse and physical, sexual, emotional, and financial forms of IPV. After statistically controlling for several correlates of IPV, they found that those who report animal abuse by their partner have an 11% increased probability of reporting they were physically and/or sexually abused (Fitzgerald, Barrett, & Gray, 2021) and a 39% increased risk of reporting emotional abuse (Fitzgerald et al., 2020). A study of those calling the US National Domestic Violence Hotline found that 37% reported their abuser had threatened to harm/kill a companion animal and 29% reported their abuser had harmed/killed a companion animal. Moreover, 50% reported they would not consider going to a shelter if they could not take their companion animal with them (Urban Resource Institute & National Domestic Violence Hotline, 2021).

The shelter-based studies and those using nationally representative samples of the general population also indicate that there is a relationship between animal abuse and controlling behavior and severe IPV. Studies have documented significant associations between animal abuse and the use of controlling behaviors (Simmons & Lehmann, 2007) and psychological abuse more generally (Ascione et al., 2007; Febres et al., 2014; Haden et al., 2018). This is supported by qualitative studies where interviewees report companion animals who they are attached to are more likely to be targeted and the threats against and abuse of companion animals employed to further control them (Allen et al., 2006; Fitzgerald, 2005; Flynn, 2000; Newberry, 2017). There are, however, important nuances. When researchers disaggregated forms of animal maltreatment they found that while IPV victims/survivors on average perceive emotional animal abuse, neglect, and threats by their abuser as premeditated and done to exert power and control over them, they are less likely to see physical animal abuse as premeditated (Fitzgerald et al., 2019).

In studies with shelter samples, animal abuse has been linked to more severe physical IPV (Ascione et al., 2007) and psychological and sexual abuse (Barrett et al., 2020). A statistically significant connection has also been documented in the general Canadian population: animal abuse is associated with a 16% increased risk of reporting being injured by one's partner and a 25% increased risk of fearing for one's life (Fitzgerald et al., 2020; Fitzgerald, Barrett, & Gray, 2021; Fitzgerald, Barrett, Stevenson, & Timmons Fritz, 2021). Research on severe IPV and femicide has also noted this connection: Walton-Moss et al. (2005) identify animal abuse as one of the strongest predictors of severe and lethal abuse. Findings from a study using data from the National Incident-Based Reporting System indicating that cases of IPV where animal abuse was also present were more likely to result in arrests than IPV in combination with other crimes may also point to a connection between animal abuse and severe IPV (Addington & Randour, 2024). That being said, in some studies, the connection between animal abuse and the severity of IPV is attenuated once sociodemographic variables are controlled for (see Tomlinson et al., 2022). Studies indicate perpetration of animal abuse and IPV is quite gendered, with perpetrators much more likely to be men (Cleary et al., 2021).

### Impacts on Abuse Victims/Survivors

The IPV literature is replete with studies documenting ways abusers isolate their victims from family/friends (Lanier & Maume, 2009). This isolation means that animals can become particularly important emotional and social support for victims/survivors (Newberry, 2017; Riggs et al., 2018; Rosenberg et al., 2020). The presence of companion animals can have important therapeutic benefits (O’Neil Hageman et al., 2018; Wuerch et al., 2017) and can even mitigate suicidality (Fitzgerald, 2007); therefore, separating victims/survivors from their companion animals can negatively impact their well-being and ability to heal (O’Neil Hageman et al., 2018).

Given these strong bonds, it is unsurprising that the presence of companion animals can impact one's willingness and ability to leave an abuser. Fifty-six percent of respondents in a sample of Canadian DV shelter clients reported that they delayed leaving their abuser due to companion animal-related concerns. Many had few alternatives to leaving their companion animal with their abuser: 60% reported that they therefore had to leave their companion animal with their abuser when they fled to the shelter. Among those respondents, approximately one-third reported they were considering returning to their abuser specifically out of concern for their companion animal. Controlling for sociodemographic variables, relationship length, and IPV severity, those whose companion animals had been severely physically abused reported a significantly higher number of unsuccessful attempts at leaving the relationship. Moreover, those who reported they had delayed leaving due to concern for their companion animal reported they had been subjected to significantly more severe IPV (Barrett et al., 2018). In a New Zealand study, 40% of respondents reported delaying leaving their abuser due to the inability to access pet-inclusive safe housing; they reported a median delay of 2 years (Roguski, 2012).

These reports are corroborated by research with DV shelter staff. In a study of staff in 17 Canadian DV shelters, 75% of respondents reported being aware of women who refused to go to the shelter specifically because they could not bring their animals. When asked about the benefits of DV shelter programs to care for companion animals, staff identified many, including that removing animals from abusers eliminates the leverage they have, decreasing the likelihood that victims will leave the shelter prematurely, and having the companion animals with the victims/survivors facilitates healing (Stevenson et al., 2018). Collectively, these findings have encouraged social policy and legislative responses to facilitate the ability of those with companion animals to terminate abusive relationships. These developments are detailed next.

## Social Policy and Legislative Responses

As awareness of these study findings has grown and frontline staff have continued to articulate the need for interventions, the number of companion animal safe-keeping programs offered by DV shelters in Canada and the United States has increased. In a recent study of DV shelters in Canada and the United States, among the 702 participating shelters, 97% reported receiving inquiries regarding assistance for their companion animals. One-quarter of the sample reported having on-site companion animal programs and 42% off-site programs (Barrett et al., 2025).

There have also been legislative efforts to address the intersecting vulnerabilities of human and animal IPV victims. In Canada, amendments to the Divorce Act in 2021 added threats and harm to animals as a form of family violence. In British Columbia, recent changes to the Family Law Act now require the court take the best interests of animals into account in determining custody, and importantly direct the court to consider if there has been family violence or a risk thereof, including abuse of or threats against companion animals. At the federal level in the United States, the Protecting Animals with Shelter Act was passed in 2018; it implemented a $2–$2.5 million per year grant fund that DV shelters and other organizations can use to develop companion animal safekeeping programs. The Act also contained a statement encouraging states to enable the inclusion of companion animals in protection orders. At the time of data collection for this article, 41 states and Washington DC had amended their protection order statutes to enable the inclusion of companion animals.

These statutes have received some scholarly attention. Articles have chronicled efforts to amend protection order statutes in specific states (Friedman & Norman, 2009; Jones, 2008), articulated state-specific recommendations (Vellucci, 2011), and provided a comparative analysis of forms of relief enabled by the statutes (Fitzgerald, 2024). Potential benefits of the statutes include: protecting IPV victims by removing animals who can be harmed/threatened in furtherance of IPV; preventing animal abuse instead of waiting for reactionary animal cruelty laws to be enforced; being more helpful than criminal prosecution; and being more responsive to the needs of victims (Mashburn, 2015; Upadhya, 2013). A study of family law professionals found that making protection order statutes animal-inclusive was one of the most highly endorsed strategies for addressing animal abuse and IPV (Fitzgerald et al., 2024).

Being able to include animals in protection orders, however, is not a panacea, as evidenced by two studies. The first examined whether states that had made protection order statutes animal-inclusive had content on the temporary and final protection order forms that specifically referenced the ability to include animals. Approximately one-fifth and one-third of the jurisdictions respectively did not (Randour et al., 2024). The second study examined the availability of DV shelter companion animal safekeeping programs in jurisdictions with animal-inclusive protection order statutes in Canada and the United States and concluded by characterizing animal inclusive-protection order statutes as a form of policy slippage because jurisdictions with these statutes are actually less likely to have companion animal programs at DV shelters in the jurisdiction. They write, “this paradox can be conceptualized as policy slippage; legislation is enacted, yet there are inadequate resources on the ground to meaningfully support the legislation” and conclude that

undoubtedly, being able to include vulnerable companion animals in protection orders is a positive step towards better protecting the human and animal victims/survivors of DV, but as it currently stands, these protection orders will only meaningfully protect those who (1) are privileged financially, and therefore are less likely to need residential DV services, and (2) those who have the good fortune to live near a shelter with a companion animal program. (Fitzgerald et al., forthcoming)

With this important caveat in mind, the current study examines the potential legal utility of animal-inclusive protection order statutes as assessed via judgments citing the statutes. Before delving into the analytical methods used, it is prudent to first address the specific problem of coercive control, where animal maltreatment can be particularly potent.

## The Problem of Coercive Control

The foregoing discussion demonstrates how understanding of the perpetration of animal maltreatment by abusers has progressed from being viewed merely as a form of property abuse to being understood as a powerful tool of abuse—coercive control more specifically. Although legally considered property, attachment to companion animals surpasses that which most people have with other forms of property. Of note, studies indicate that animals who IPV victims/survivors are more attached to are more likely to be mistreated by their abuser (Faver & Strand, 2003).

This instrumentalization of animal abuse in furtherance of IPV is consistent with feminist theories that conceptualize IPV as motivated by the desire to establish coercive control over women, which can even be normalized in patriarchal societies (Adams, 1995; Dutton & Goodman, 2005; Johnson, 2008). As part of that process, abusers are said to focus on their victims’ vulnerabilities, and companion animals can be a potent vulnerability. For instance, one study documented cases where abusers used threats and companion animal abuse to coerce their human victims into engaging in illegal behavior, which then gave the perpetrator further leverage in the form of threatening to expose that behavior as well (Loring & Bolden-Hines, 2004).

Academics, practitioners, and policymakers alike have paid greater attention to coercive control in recent years. Understandings and operationalizations of the concept vary (Barlow & Walklate, 2021), but the following succinct description by Stark (2007, p. 205) is instructive: the combination of coercion and control produces a “condition of unfreedom.” Feminists have been at the forefront of focusing on this type of nonphysical abuse, which receives less sociolegal attention than physical abuse even though survivors often speak to how nonphysical abuse impacted them just as much, or even more than, physical abuse (McMahon & McGorrery, 2020).

The empirical research indicates that higher levels of coercive control are associated with increases in the risk of severe physical IPV; moreover, coercive control can persist long after the victim has separated from their abuser. Upon evaluating the research in the field, Stark and Hester (2019, p. 91) conclude it “reinforces our belief that coercive control by a male partner is the most common and most devastating context for women's help-seeking because of abuse … prior level of control predicts post-separation violence/sexual assault and fear of mediation. These findings support the importance given to the ‘level of control’ in risk models of femicide.”

Growing recognition of risks posed by coercive control and the fact extant laws are limited in their ability to respond to this type of nonphysical abuse have facilitated the enactment of new offenses criminalizing coercive control, such as in England, Scotland, the Irish Republic, Northern Ireland, and Tasmania (for a legislative overview and critiques of these developments, see Stark & Hester, 2019). Efforts such as these acknowledging the impact of coercive control have required taking account of abusive behaviors above and beyond physical abuse, and “demands that practitioners, and the tools at their disposal, take account of a course of conduct” (Barlow & Walklate, 2021, p. 892) instead of remaining incident-focused.^2^

Protection orders are one such tool, which depending upon the jurisdiction, can be both criminal and civil. These orders have been one way to work around the limitations of relying on criminal law—which has been physical abuse and incident-focused—to address abusive behavior. Protection orders are said to

provide scope to better capture the ‘lived experience’ of IPV, such as the broad range of behavior that might ground an order, the consideration in some jurisdictions about how acts and behaviors function (e.g., to cause fear), the lower standard of proof, the focus on future protection…. (Wangmann, 2020)

Because they are, at least in theory, not incident-based, applicants can provide a history of harmful behavior, enabling the identification of patterns and a greater understanding of their fear.

Increasing awareness that abuse can extend beyond the physical and nonetheless be extremely harmful and serve as a marker of heightened risk, particularly postseparation, likely created an environment more conducive to amending protection order statutes to enable the inclusion of animals. The degree to which these statutes can assist in mitigating coercive control, however, has not yet been examined and is addressed in the current study.

## Method

Specific keywords were used to identify relevant case law in the United States using West Law. The animal-inclusive statutes in 41 states and Washington DC were all entered into separate searches in combination with the following keywords: animal, pet, dog, cat, rabbit, or horse. This method identified written judgments in 50 cases. Written judgments are provided when the court adjudicates a case by opinion; it explains the formal decision arrived at by the court (Kumar et al., 2013). The year 2022 is the most common publication year of these judgments, which spanned across 18 years and are from the following 18 states: Arizona (1), Arkansas (1), California (8), Connecticut (2), Delaware (1), Florida (4), Kentucky (1), Massachusetts (4), Michigan (4), Minnesota (2), Missouri (2), Nebraska (1), New Hampshire (2), New York (10), North Carolina (1), Ohio (1), Tennessee (2), Vermont (1), Washington DC (1), and Wisconsin (1). Six pages is the median number of pages in each judgment and a total of 379 pages were analyzed across all judgments. Twelve of these cases focused on animal concerns outside the specific context of gender-based violence and were therefore removed. The analysis in this article focuses on the 38 cases that cite the animal-inclusive protection order statutes specifically in the context of gender-based violence.

Three rounds of coding were used to identify themes in the judgments, some of which were developed deductively from the literature, while others emerged inductively from the data (see Braun & Clarke, 2006). The first round of coding involved an initial reading of the judicial decisions and the construction of coding categories. Next, the judgments were coded again to ensure consistency across codes and cases. Finally, a third round of coding was used to further refine by collapsing codes and developing themes. This type of thematic analysis has proven successful in other studies examining judicial decisions (e.g., Kammeringer, 2023). The nature of the data does present a methodological limitation: the study is restricted to cases where written judicial decisions are available, thus in most instances, these are cases that went to appeal.

## Findings

All cases were coded as belonging to at least one of the following four categories based on the type of harm it centered on: IPV (24 cases), child abuse (nine cases), stalking/harassment (15 cases), and wrongful imprisonment (one case). Several cases fell into more than one of these categories (e.g., IPV and child abuse), and the categories are therefore not mutually exclusive.

In most cases, information was provided regarding reasons for including animals in the protection order. However, seven of the 49 instances coded as addressing IPV, child abuse, stalking, and/or wrongful imprisonment merely noted that animals were included in the protection order without providing details. Moreover, in three other cases, the addition of animals to the state's protection order statute was referenced, but specific instances of animals being included in protection orders were not addressed. For instance, in Kings County, New York v. R.H., A. B. & J. P (2010), the court held that foster care agency staff were ineligible for a protection order against a child's parent because they are not referenced in the statute as a group that can be protected. The court pointed to the addition of companion animals to the protection order statute as evidence that when the legislature wants to extend protections to a group, they amend the statute accordingly. In Ram-Parker v. Parker (2009), the court cited the inclusion of animals in the state's statute as evidence that the state is taking IPV seriously.

This section focuses on the judgments where details vis-à-vis animal and human maltreatment, interests, and well-being were provided, organized around four themes: connections between the victimization of people and animals and their mutual well-being; animal abuse as grounds for a protection order; the role of animal-inclusive protection orders in mitigating coercive control; and the relevance of animal-inclusive protection orders to habeas corpus applications where IPV is alleged.

### Connections Between the Victimization of People and Animals and Their Mutual Well-Being

In 12 cases, the courts explicitly described and interpreted animal and human abuse as being connected. In Brown v Brown (2020), for instance, a father appealed a custody order awarding the children's mother sole custody. One matter raised upon appeal was that the father's abuse of the family's companion animals had been included in evidence as grounds for awarding sole custody to the mother. In their decision, the appeals court ruled that the evidence was pertinent:

Father's abusive treatment of family pets was properly relied upon by trial court as a basis for modification of child custody to grant mother sole legal and physical custody of children; there was evidence that plaintiff threw a family dog against the wall for chewing on shoes, and father admitted that he kneed another dog in the chest for stealing food, and shot an airsoft pistol at a cat that was on the counter.

They also noted “his mistreatment of the family pets perpetuated a fearful environment to compel good behavior, and although smoking and drinking [by mother] may not have been healthy behaviors to model for a child, violent and cruel behavior was a far more serious moral failing” (p. 4), clearly articulating a connection between animal abuse and child well-being.

The connection between human and animal victimization and well-being was also addressed in stalking cases. In PDJ v SS (2017), the respondent appealed a protection order issued against him. He argued there was insufficient evidence of stalking to warrant a protection order. The appeals court disagreed, pointing to two incidents they argued constituted a “course of conduct” (p. 4) sufficient to warrant a protection order for the applicant and her dogs. One involved vandalism of her car and the other a note left warning her to keep her dogs quiet “or else.” In this case and others, threats and animal maltreatment were relied upon as evidence of harmful behavior inflicted upon people and indicative of risk for future harmful behavior.

The legal significance of having animal abuse included in definitions of family violence in protection order statutes and viewing these forms of harm as interconnected is further evidenced in two criminal cases. In People v Jones (2010), the defendant appealed a conviction for child endangerment and cruelty to animals for stabbing his girlfriend's 10-year-old son's dog to death. The defense argued that animal abuse (killing the dog and previous incidents of hitting cats with bats) should not be taken into consideration vis-à-vis the child endangerment charge. The appeal court disagreed, and in its decision affirming the conviction, wrote

killing Jack [the dog] constituted behavior that could have been enjoined under Family Code section 6230. It therefore constituted DV under Family Code section 6211, and also under Evidence Code section 1109, subdivision (a)(3) … We therefore conclude that the evidence of prior acts of DV was admissible. Moreover, the trial court did not err by instructing the jury that it could consider the prior acts of domestic violence as evidence of a propensity to commit the acts of child endangerment alleged in this case. (pp. 5–6)

In People v. Kovacich (2011), the defendant appealed his conviction for first-degree murder in the death of his wife. Some of the appeal arguments focused on the defendant's treatment of the family dog and whether statements regarding that treatment should have been entered into evidence at trial. The defense argued evidence related to statements the victim made to friends and family that the defendant kicked the dog to death, as well as his own admission of animal abuse to police, should be inadmissible as evidence of IPV, were prejudicial, and “appear[ed] to be a way by the prosecution to introduce proscribed character evidence barred under [section] 1101” (pp. 19–20).

The appeals court upheld the trial court's decision to allow the evidence, reasoning:

the question remains as to whether evidence of the incident in which defendant violently kicked the family dog amounts to an act of abuse against Janet … If it does, then it is admissible not only under section 1101 to prove his motive, but also under section 1109 to prove his propensity to commit the murder. For the following reasons, we hold that it does … Family Code section 6320 authorizes the court to issue a protective order regarding ‘any animal owned, possessed, leased, kept, or held by either the petitioner or the respondent or a minor child residing in the residence or household of either the petitioner or the respondent,’ and further authorizes the court to enjoin the respondent from ‘molesting, attacking, striking, threatening, harming, or otherwise disposing of the animal…’ Thus, defendant's assault on the family dog amounted to ‘abuse’ within the meaning of Family Code section 6203. This abuse was committed against his wife and children, who witnessed the violent assault, and amounted to ‘domestic violence’ within the meaning of Family Code section 6211. (pp. 21–22)

The court further explained in a footnote that expert witness testimony admitted into evidence was warranted because “without this testimony, the jury might not have understood that abusing an animal is taken to be a form of threat to the victim, which would cause the victim to be afraid of leaving the relationship. Thus the trial court did not abuse its discretion in allowing this testimony, and the prosecutor did not engage in misconduct by eliciting it” (p. 28).

### Animal Maltreatment as Grounds for a Protection Order

In that same case—and in 10 others—the judgments also described animal abuse as motivation for seeking, and as grounds for issuing, a protection order. In People v. Kovacich (2011), the appeals court decision recounted how the victim had reportedly decided to leave her husband and considered obtaining a protection order specifically because he allegedly killed the family dog:

Janet believed that Fuzz's death was caused by the kicking. In tears, she told her brother Gary about the dog's death and explained that ‘she was starting to feel threatened at home, and she was worried for her safety, and she was worried for [their children]…’ Janet cried as she told … the wife of another Placer County Sheriffs’ Department sergeant [Defendant was a K9 Police Officer], that defendant had kicked the dog to death and that she was ‘very frightened’ of defendant. She also told … two of her neighbors, that defendant had kicked the dog to death. [They] described her demeanor as ‘very sad and very upset…’ ‘hysterical, crying, extremely distraught.’ (p. 8)

In Hart v. Bernhagen (2022), the applicant testified her husband's actions toward family dogs factored into her decision to obtain a protection order. The appeals judgment describes how her husband

grabbed his pistol and tried to shoot one of the dogs, grabbed a second dog by the collar and held him up in the air, choking him, and attempted to get a third dog to attack the second dog. Hart testified, ‘And the entire time I was screaming at him to let go’ … Bernhagen eventually dropped the dog, which revived, but Bernhagen continued to be aggressive … Bernhagen again shook their child in his high chair following the dog incident, and that he also approached her, told her to ‘put [her] mouth on it,’ and when she declined he ‘dragged [her] by [her] hair over a lawnmower, pulled down [her] pants, and forced himself on [her] after the entire thing…’ her decision to file a protection order was ‘motivated through the fact that [Bernhagen] almost killed two dogs, shook [her] son, and forced himself on [her].’ (p. 3)

In other cases, animal maltreatment was referenced specifically as evidence in support of issuing a protection order and/or that a protection order had been violated. In NS v BS (2022), the husband appealed a protection order ordering him to stay away from his wife, children, and companion animals. The appeals court upheld the order, noting that his daughter testified that “He threatened to abuse her animals…” and the wife “also testified that Husband threatened to shoot their animals” (p. 7). In another case (Winkler v Winkler, 2011), abuse of animals outside of the home (shooting at neighbors’ cats) was entered into the record as one aspect of the defendant's behavior warranting a protection order. In AB v SB (2024), yelling at the petitioner's dog (who was included in the protection order) when the petitioner was walking him was explicitly referenced as evidence the respondent had violated the protection order.

In contrast to those cases where animal maltreatment was relied upon as evidence, there were two instances where the courts explicitly bracketed out potential harm against animals. In Simmons v Dixon (2006), an ex-boyfriend appealed a protection order granted to his ex-girlfriend who alleged that he had threatened to kill both her and her dog, and the appeals court affirmed the protection order. However, the court decided not to take up the issue of whether the threats against her dog were relevant, and instead asserted that “it is clear that the trial court did not grant relief based on Simmons's threats to the dog, but rather his threats to ‘beat’ Dixon herself.” In Tate v Tate (2018), a brother appealed a decision granting his sister a protection order against him after he broke into her house and took her cats. The appeals court overturned the decision, arguing there was insufficient evidence that the sister had grounds to fear her brother would harm her and could not demonstrate that he was a threat to the animals either.

### The Role of Animal-Inclusive Protection Orders in Mitigating Coercive Control

In several cases, animal abuse was referenced in judgments in a manner that enabled understanding it as part of coercive control. Koehn v Holley (2016) is illustrative. A protection order was issued protecting a woman and her dog; the respondent appealed, arguing there was insufficient evidence for a protection order. The appeals court disagreed, affirmed the protection order, and provided significant detail regarding how the defendant was fixated on the dog. In a text message to the plaintiff quoted in the judgment, the defendant stated,

I have plans to as many legal actions as necessary to locate Cody [Plaintiff's dog]. This could become very embarrassing and very expensive for whoever has him … I will get Cody back regardless of what it takes. [I] would take this very seriously. This is not a bluff. Love, Wayne.

There were other cases where the state's animal-inclusive protection order statute was invoked as enabling the court to consider harm or threats against parties beyond the individual applying for the protection order. In Fisher v. Minichiello (2007), the ability to include animals in protection orders was cited to demonstrate that stalking can extend beyond the individual target to others in their life, and that courts can consider that behavior part of the stalking. In this case, an administrator of an assisted living facility requested and was granted a protection order against a patient's family member who had been harassing her and her staff. In another case (LC v WC, 2021), the appeals court denied an appeal of a protection order granted to a woman to protect her from her husband. The husband argued that his pointing a gun at his wife's aunt should not be used as evidence of IPV because his actions were not perpetrated directly against his wife, who was not home at the time. The court explained in its decision that the law

does not require the specified criminal conduct to be directed at the plaintiff. Indeed, the statute provides that the commission or attempted commission of ‘[c]ruelty to animals as defined in RSA 644:8’ may constitute ‘abuse’ … if an act of cruelty to an animal can pose a credible, present threat to a plaintiff's safety, then an act directed at a plaintiff's aunt can also pose such a threat.

The inclusion of animals in protection orders was a specific point of contention for some respondents/defendants: there were seven cases where the inclusion of animals was appealed. In Simmons v Dixon (2006), Simmons appealed a protection order protecting his ex-girlfriend and her dog. He admitted threatening to kill the dog but argued he would not have followed through. He asserted on appeal the dog should not have been included in the protection order, arguing

Dixon's only fear of ‘imminent’ harm related to [his] name-calling and alleged threats to the dog he bought her while they were intimately involved. Simmons claims that the legislature did not intend the protective order scheme to apply to family pets.

The appeals court disagreed, affirming the protection order. In another case, where a woman and her dog were included in a protection order and the inclusion of the dog was appealed, the court was unpersuaded by the argument that the trial court had erred in including the dog because the judge “did not particularly mention one of the activities enumerated in the statute.” They concluded, “While the judge did not specifically cite one of the statutory examples, it is apparent from the record that the plaintiff's request was to prevent the defendant from ‘interfering with’ her dog … There was no error in extending the protective order to the plaintiff's dog” (Portanova v Orme, 2016).

In other cases, the respondent appealed both the inclusion of animals as well as people other than the applicant herself. In Tronson v Ortiz (2022), the respondent appealed the inclusion of his ex-girlfriend's family members and her dog in the protection order. The appeals court affirmed the order. In BR.C. v BE.C (2024), Sophy v Voss (2023), and Waters v Waters (2022), the respondents/defendants appealed the inclusion of their children and animals in the order. In all three cases, the appeals courts affirmed the protection orders. In Waters v Waters (2022), the appeals court specifically noted that it did not matter who owned the cat in question:

We reject Charles's argument that the district court lacked authority to dispossess him of Anita's son's cat. The district court may ‘direct the care, possession, or control of a pet or companion animal owned, possessed, or kept by the petitioner or respondent or a child of the petitioner or respondent….’ This is so regardless of whether Anita's son is the cat's owner.

The statute in the jurisdiction (Minnesota) is worded such that legal ownership of an animal need not be determined to include him/her in a protection order—as the court noted, it did not matter if the cat was legally owned by the child in question. The wording of the protection order statute can therefore be critical in determining how well coercive control vis-à-vis animals can be mitigated.

In other states, the statute wording is insufficient to preclude the use of animals as a mechanism for coercive control. In Stone v Town of Cicero (2024), a woman had secured a protection order for herself and her dog against her ex-husband. In that jurisdiction (New York), however, the wording of the statute only enabled restricting her ex-husband from harming the dog. During an altercation, the woman saw the dog in her ex-husband's truck, and when the police officer at the scene failed to retrieve the dog, she tried to do so and suffered a dislocated shoulder when the officer tackled her upon failure to comply with his order to stay away from the truck. She consequently sued the officer and the town, arguing he had “negligently failed to enforce the Order of Protection” related to the taking of her dog and “repeatedly ignored conduct by Mr Douglas that was in direct breach and violation of the Order of Protection occurring in his presence.” The appeals court dismissed her claim, explaining “the Order of Protection does not expressly prohibit Mr Douglas from taking Rex, only that he may not injure or kill Rex….”

Of all the judgments analyzed, there was one case (Kollaman v Caudill, 2023) where the inclusion of an animal in a protection order was overturned; the case involved nontraditional companion animals. The Circuit Court ordered the woman petitioner could keep the kangaroo, zebras, and camels the couple had. The ex-boyfriend appealed and argued that because they both were animal trainers and operated a petting zoo, the animals were kept for “bona fide agricultural” reasons, which exempted them from being considered “family pets” and therefore could not be included in the order. The appeals court agreed and struck their inclusion.

### Animal-Inclusive Protection Orders and Habeas Corpus Claims on Behalf of Abused Women

In 2006, a writ of habeas corpus was brought on behalf of Rena Alspaw. It asserted that Alspaw had been wrongfully imprisoned for first-degree murder of her ex-boyfriend in 1996. The grounds for the application included that in the ensuing years, the expert witness who had diagnosed the defendant with PTSD and battered child syndrome, but not battered woman syndrome (which would have made available a battered woman defense), had changed her opinion. The psychiatrist had not diagnosed Alspaw with battered woman syndrome because, at the time, the scope of behavior considered to constitute violence in a relationship was narrower. Since then, the academic literature has evolved and the stalking the defendant had been subjected to was increasingly being viewed as a form of abuse warranting consideration. Likewise, animal abuse was increasingly being considered salient vis-a-vis IPV.

In Alspaw's original trial, the court ruled that the deceased's convictions for animal cruelty could not be admitted into evidence. The expert witness was given access to the police reports that detailed his animal abuse after the trial. These documents, she argued, provided evidence the deceased had “displayed exceptionally pathological potentials” and engaged in “unspeakable acts toward defenseless animals.” She concluded, “it is difficult to see how this is not relevant to the effect he had on a teenage girl from an abusive background.” The expert witness further explained in support of the petition that in the years since the conviction, she had come to understand that “there is a clear tie [of animal abuse] to the dynamics of perpetrators of DV,” and knowing someone is capable of violence with animals would “definitely [be] in the back of a battered woman's mind.” The trial court found this persuasive and granted the writ of habeas corpus, vacating the conviction.

The state (California) appealed the decision, and ultimately the appeals court ruled in its favor. The state argued the victim's convictions for animal abuse should not be considered because

evidence of specific acts by the victim tending to show his character for violence is not reviewable under section 1473.5 … Swanson's abuse of animals did not fall within the definition of domestic abuse … for which Dr. Kaser-Boyd's testimony would have been admissible. (In re. Alspaw, on Habeas Corpus, 2010)

Indeed, at the time of the case, animal abuse was not included in the state's law as part of IPV, nor animals a potential party to be protected in a protection order. They were, however, added a few years later in 2015 (California Family Code).

## Discussion

The findings provide evidence in support of affirmative responses to the following research questions examined here: (a) Is there evidence in judgments that the courts are taking the inclusion of animals in protection orders seriously and viewing animal abuse as interconnected in important ways with gender-based violence? (b) Are animal-inclusive protection order statutes being interpreted in a manner that provides protection for human and animal victims, particularly from coercive control? There are several significant implications, which are attended to in this section, as are points of caution and aspects in need of further research.

### Animal-Inclusive Protection Orders: Protecting Human and Animal Victims

The cases analyzed provide substantial evidence that animal-inclusive protection order statutes are being cited in judgments in a manner assistive to both human and animal victims of abusive behavior and therefore provide support for the working assumption that “the addition of pets to protection orders expands the potential effectiveness of protection orders to reduce harm to domestic violence survivors” (Randour et al., 2024, p. 3441). There were many instances where significant animal abuse was noted in judgments, and accordingly, the inclusion of animals in protection orders likely prevented further animal abuse. There were also many judgments where the courts explicated connections between human and animal victimization and well-being. Of note, there were cases where the courts used animal maltreatment as evidence warranting a protection order, and in others, animal abuse was described as a motivator for seeking an order. There was also substantial evidence of animals being (ab)used in ways consistent with coercive control, discussed below. Therefore, the ability to include animals in protection orders has likely also protected human victims. By and large, the judgments framed animal maltreatment and well-being as serious considerations, and implicitly not reducible to property abuse.

Theoretical and empirical scholarship has suggested animal maltreatment and gender-based violence so commonly co-occur because threats/harms to animals can be instrumentalized by perpetrators to harm human victims and establish coercive control (see Fitzgerald, Barrett, & Gray, 2021; Fitzgerald, Barrett, Stevenson, & Timmons Fritz, 2021 for a review). Legal interventions aimed at addressing and mitigating coercive control, however, have been fraught with challenges. Coercive control laws can be difficult for police to enforce and the criminal justice system to punish (Stark & Hester, 2019). Animal-inclusive protection order statutes appear assistive in mitigating these challenges. The findings indicate they are useful for (a) minimizing the extent to which coercive control can continue postseparation vis-à-vis companion animals, and (b) expanding the scope of evidence courts can take into consideration in awarding protection orders and assessing if they have been violated.

Regarding the former, the ability to include a companion animal in a protection order affords animals protection and removes them as instruments of coercive control. Many judgments analyzed referenced significant companion animal abuse. It seems reasonable to assume said abuse would have continued if the aggressors were not restricted from accessing the animals. The impact of being able to include an animal in a protection order is therefore likely important for the protected animals and those who care for them, particularly given research indicates animal abuse is a particularly powerful mechanism for coercive control.

Moreover, attending to animal well-being can expand the scope of evidence under consideration for a protection order and assist in shifting from incident-based responses to considerations of broader patterns of abuse. Protection orders in general enable taking the broader context of abuse into account more than criminal charges do because the focus tends to be discrete incidents. Nevertheless, there are concerns that the legal system can default to focusing on incidents when considering protection order applications, thus obviating the broader context (Wangmann, 2012). The findings detailed herein indicate that considerations regarding animals in protection orders can provide a useful avenue for accounting for the broader context of abuse and moving beyond focusing on specific instances of physical violence. The judgments were replete with descriptions of ways animals were threatened and harmed, and the perception that abuse was part of the constellation of coercive controlling behavior; that is, behavior that does not target the human victim physically and instead extends beyond her to target those she cares for. As noted in a decision in one of the stalking cases analyzed, threats against animals can serve as evidence of a “course of conduct”; without that evidence, an order might not be issued.

The efficacy of animal-inclusive protection orders in addressing and mitigating coercive control is perhaps most clearly demonstrated by the seven cases where respondents/defendants specifically appealed the inclusion of animals. It would seem these individuals considered gaining access to the protected animals worth the time and money involved in appealing these orders, which is likely partially—if not entirely—related to their value in coercive control.

### Loopholes Identified

Only one appeal of the inclusion of animals was successful: Kollaman v Caudill (2023) involved a case where nontraditional companion animals (i.e., kangaroo, zebras, and camels) were involved. The appellant successfully argued these were not companion animals and were more appropriately viewed as kept for “bona fide agricultural” reasons for the couple's petting farm and animal training business. The divergence in opinions between the lower and appeals courts in this case is indicative of the challenges of placing animals into discrete categories and raises important questions. On what basis should animals be categorized as companion animals versus agricultural animals? Based on species? Level of human attachment to them? Capital generated through their use? It is also unclear how service or emotional support animals would be categorized, particularly those who do not belong to the most common types of companion animal species. Regardless, this case points to a loophole in animal-inclusive protection order statutes that extend only to companion animals and reference exemptions for agricultural animals. Those animals can nonetheless also be mistreated and used as a mechanism for coercive control.

Another loophole vis-à-vis the relief that can be ordered under animal-inclusive protection orders is elucidated herein. Stone v Town of Cicero (2024) points to limitations of statutes that merely prohibit harming animals, but not contact (some states do ban contact; see Fitzgerald, 2024 for a description of the types of prohibitions). In this case, a woman was injured when she attempted to secure her dog from her ex-husband; she was under the (reasonable?) impression that all contact with her dog—not just harmful contact—was prohibited.

In an earlier analysis focused on animal-inclusive protection order statutes by the author of the current study (Fitzgerald, 2024), a third potential loophole in the wording of some statutes was identified: some only cover animals legally owned by the petitioner. In one of the analyzed cases (Waters v Water, 2022), the legal ownership of the animal in question was challenged. The husband/father appealed the inclusion of a cat in the protection order, arguing that “the district court lacked the authority to dispossess him” of the cat. Referencing the statute, which in the State of Minnesota (2020) goes beyond ownership to include animals “possessed or kept,” the appeals court disagreed and kept the cat in the protection order. If this case had occurred in a state that only enables the inclusion of animals legally owned by the petitioner (and/or children in the home), the cat presumably would have been excluded. The wording of these statutes vis-à-vis ownership can be a matter of life and death. Focused research in jurisdictions with ownership-based wording in their protection order statutes would be helpful to elucidate cases where animals are excluded because of an ownership claim by the abuser; such research could provide additional evidence of the need for statute amendments.

### Enabling More Expansive Considerations for Human Victims

Despite these loopholes, the findings indicate that animal-inclusive protection order statutes are enabling protection against harassment and coercive control, even above and beyond the use of animals. In two cases, the courts used the statute as evidence that stalking can and does extend beyond the specific target to those in their life, such as coworkers (Fisher v. Minichiello, 2007) and aunts (LC v WC, 2021)—thus making it possible to address tactics of harassment and coercive control that extend beyond the specific target of the abuse. In another case (Kings County, New York v. R.H., A. B. & J. P, 2010), however, the affirmative inclusion of animals in the state's protection order statute was used to deny more expansive inclusion of other parties under an order; the court cited the statute as evidence that if the legislature intends more expansive protections (i.e., to foster care agency staff), they amend the statute accordingly (i.e., the inclusion of animals). Thus, while the inclusion of animals in protection order statutes has been used by some courts to expand the ability to take a pattern of conduct in harassment and coercive control into account, in the state of New York it has also been used to deny expansion of protections.

There were also two instances where the inclusion of animal abuse in definitions of IPV and adjoining protection order protections were used to expand what courts could consider as evidence in criminal cases. In cases involving child endangerment (People v Jones, 2010) and homicide (People v Kovacich, 2011), the stakes were high in admitting animal abuse as a form of DV. Both defendants appealed their convictions, arguing that animal maltreatment should not have been admitted and was prejudicial. In both cases, the animal-inclusivity of protection order statutes and definitions of family violence were cited as making animal abuse relevant and the appeals were dismissed. It is unclear if these forms of utilization of animal-inclusive protection order statutes were considered by legislators when they enacted them—research examining deliberations over the bills would be required to speak to this. That consideration notwithstanding, several scholars have voiced concern regarding carceral responses to animal abuse (e.g., Beirne, 2009; Marceau, 2019); to the best of my knowledge, however, the potential evidentiary impacts of including animal abuse in definitions of DV and animals in protection orders has not been specifically addressed prior to this article.

Whether or not this is a positive development depends upon one's perspective. Undoubtedly, there is no simple answer. On the one hand, taking animal maltreatment into account can assist with moving beyond incident-based, physical abuse-focused abusive behavior and facilitate more fulsome considerations of coercive control—something scholars have been urging the legal system to do. However, it is important to acknowledge that it could also facilitate criminal justice responses that may not necessarily reduce abusive behavior in the long term, a point critical sociologists/criminologists and anticarceral feminists have been making for decades (e.g., Christie, 1993; Snider, 1998). Space constraints prevent me from wading deep into this debate here, although I would like to offer a few general observations.

The impact of animals (and their abuse) on the legal system is likely just beginning to unfold. It is being facilitated by many factors, including increasing numbers of people sharing their lives with companion animals, more animals and their abuse coming under the consideration of the court, and more animals engaged in work within the criminal justice system. Take for instance a case a few years ago where a child abuse conviction was appealed because the child witnesses were accompanied by a therapy dog as they testified in court. The defense argued the dogs’ presence may have prejudiced the jury in favor of the children and against the defendant (Coren, 2024).

We might also consider if the presence of animal abuse may also prejudice a jury, or judge for that matter, against a defendant. It is a somewhat ironic situation: animals are legally considered property, yet they are viewed by many as the epitome of vulnerability and innocence, and their abuse (of companion animals at least) abhorrent. As an increasing number of homes contain companion animals, and empathy for them and intolerance for their abuse grows by extension, their impact on the legal system and (perceived) impartiality can be expected to as well.^3^ Indeed, there has been at least one instance to date where a justice recused himself from a dog abuse case because of a self-declared affinity for dogs (CBC News, 2015).

On the other hand, acknowledging that animal abuse is important to consider in the context of IPV could also be assistive to some in conflict with the law, specifically those accused of killing their intimate partner due to alleged abusive behavior. In the Alspaw (2010) case, the trial and final appeals courts ruled that animal abuse perpetrated by the ex-boyfriend she killed could not be entered into evidence because it could be prejudicial and because at the time it did not fall within the state's definition of IPV. The invocation of animal abuse perpetrated by the deceased as part of a battered woman defense is not unique to this case.

There have been at least two other cases where animal abuse was raised as part of a defense for women who killed their allegedly abusive partners. In 2018, a woman in Texas shot and killed her husband after he beat the family cat. She stated she shot him in self-defense because she feared that he was going to abuse her next. She received 5 years’ probation (NewsRadio 1080 KRLD, 2022). In another cat-related case, this time in British Columbia, Canada, a young woman killed her boyfriend the morning after he choked her cat. She was charged with first-degree murder. At trial, her attorneys mounted a battered woman's defense, arguing with the assistance of an expert witness that the abuse of the cat needed to be understood as part of the abuse he had perpetrated against her. She was instead convicted of second-degree murder (Nanaimo News, 2024). At the time of this writing, sentencing is still pending.

It is unknown how many other cases there have been where animal abuse has been considered relevant to self-defense due to battered woman's syndrome, or how many times it has been denied and women incarcerated as a result, such as Alspaw. It is also uncertain if animal abuse had been included in the definition of IPV and animals a party that could be protected in protection orders at the time of the appeal of Alspaw's successful habeas corpus petition if her conviction would have ultimately been vacated. Given the court noted in its decision that animal abuse was not part of the definition of IPV at the time (it was added a few years later), and therefore it was outside of the scope of the case, it would seem it would have made a difference.

Animal-inclusive protection order statutes could therefore end up being a useful mechanism for enabling the inclusion of animal abuse in considerations of self-defense. In acknowledging the interconnections between animal maltreatment and IPV, particularly how animal abuse can be part of coercive control and how it can heighten fear for one's own life, these statutes could be assistive in defending IPV victims/survivors who kill their abusers. Further research is needed to explore the potential depth and scope of these legal implications.

In addition to the Alspaw case, there were two other cases (Simmons v Dixon, 2006; Tate v Tate, 2018) where the judgments indicate the courts explicitly decided not to take up the issue of animal maltreatment. In another case (Moreno v Beltran, 2020), a requested protection order to protect an animal (but not people as well) failed. In this case, the applicant was identified as a man and the respondent a woman, so there may be other confounding factors here above and beyond potential unwillingness to consider the animal in question's well-being. Nonetheless, these cases stand out as exceptions to the general trend documented here of courts taking animal maltreatment and its intersection with human well-being into serious consideration.

## Conclusions and Recommendations

The evidence indicates that in the majority of cases, animal-inclusive protection order statutes were interpreted in ways conducive to protecting human and animal victims of gender-based violence; most specifically, the findings indicate that these orders are a useful tool for appreciating and mitigating the impacts of coercive control, although some important loopholes were identified. Specific recommendations for extending these protections are provided below.

Given the documented value of animal-inclusive protection orders for protecting human and animal victims/survivors, I recommend that U.S. states that have not yet amended their protection order statutes to enable the inclusion of animals do so without delay. Yet it is important to acknowledge that the United States has been at the forefront of expanding protection order statutes to include companion animals. The findings ought also serve as encouragement for jurisdictions in other countries to do so as well. For instance, to date, there are only a couple of provinces in Canada that explicitly enable the inclusion of companion animals in protection orders. There is much room for improvement there.

The findings also indicate the wording of the statutes is critically important: those that enable the inclusion of animals legally owned by the defendant/respondent provide the broadest and deepest levels of protection. Therefore, I suggest jurisdictions that do not yet have animal-inclusive protection order statutes draft their statutes with that in mind. Furthermore, I think it prudent for jurisdictions that currently only allow the inclusion of animals legally owned by the applicant and/or children in the home (see Fitzgerald, 2024 for a detailed list) to revise their statutes accordingly. Also, careful thought needs to be given to wording that exempts agricultural animals and other nontraditional companion animals. This exclusion could put those animals and the people who care for them at risk.

Although the findings indicate animal-inclusive protection orders are useful in providing victims/survivors with important legal recourse to protect themselves and their animals, other steps to ensure their safety may nonetheless be circumscribed. In one study, animal-inclusive protection order statutes are described as an instance of policy slippage because DV shelters in jurisdictions in Canada and the United States with these statutes are less likely than those without companion animal safekeeping programs that enable survivors to flee their abuser with their companion animals (due to a number of potential factors, not because DV shelters view animal-inclusive protection orders as solving the problem; Fitzgerald et al., forthcoming). This particularly disadvantages those who do not have resources to support themselves and are therefore more likely to need DV shelter services. Therefore, while being able to include companion animals in protection orders is an important legal step, it is also critical that jurisdictions have programming in place to provide victims/survivors with alternatives to staying in abusive relationships for the sake of their companion animals or leaving their companion animals with their abuser. Domestic violence shelters are underresourced, and therefore, more expansive development of these life-saving programs will require the provision of additional, targeted resources.

There was a noteworthy case among those analyzed where the courts were asked to consider animal abuse part of a constellation of factors that would make a battered woman's defense available for killing one's abusive partner. In the case, the court ruled against it because animal abuse was not part of the definition of IPV in the state (it was subsequently added to enable animal-inclusive protection orders). As noted, there have been other cases where animal abuse has been invoked as critical to a battered woman's defense. While attention has been paid to the potential of expanding criminal defenses so that threats or harms to animals can be addressed as more than mere threats/harm to property (Marceau, 2015), attention should be paid specifically to including animal abuse as a legal consideration under a battered woman's defense. Given (a) the importance of companion animals to some, especially victims/survivors, (b) the empirically documented connection between animal abuse and IPV, and severity specifically, and (c) the fact that in the majority of states animals can be included in protection orders, it is time to explore adding animal abuse as a consideration under the battered woman's defense.

## Notes

1. McKinney's Family Court Act § 842 passed in 2006.

2. It is worth noting that 10 U.S. states have included animal maltreatment in their statutory definitions of coercive control (Urban Resource Institute & National Domestic Violence Hotline, 2021).

3. In one of the cases analyzed here (NS v BS, 2022), the defense actually called witnesses specifically to testify that they had never witnessed the defendant harm animals.
